# Prefrontal and ventral striatal neural correlates of reversal learning in anorexia nervosa and bulimia nervosa

**DOI:** 10.3758/s13415-025-01370-5

**Published:** 2025-11-26

**Authors:** Matthew F. Murray, Brianne N. Richson, Glen Forester, Neil P. Jones, Elizabeth N. Dougherty, Angeline R. Bottera, Lisa M. Anderson, Lauren M. Schaefer, Erika E. Forbes, Jennifer E. Wildes

**Affiliations:** 1 Department of Psychiatry and Behavioral Neuroscience, University of Chicago, 5841 S Maryland Ave., Chicago, IL MC307760637, USA; 2 Charlie Health, Inc., Bozeman, MT, USA; 3 Center for Biobehavioral Research, Sanford Research, Fargo, ND, USA; 4 Department of Psychiatry and Behavioral Science, School of Medicine and Health Sciences, Department of Psychiatry, University of North Dakota, Grand Forks, ND, USA; 5 Department of Psychiatry, University of Pittsburgh School of Medicine, Pittsburgh, PA, USA; 6 Life Span Institute, University of Kansas, Lawrence, KS, USA; 7 Department of Psychiatry and Behavioral Sciences, University of Minnesota Medical School, Minneapolis, MN, USA

**Keywords:** Anorexia nervosa, Bulimia nervosa, Reversal learning, Functional magnetic resonance imaging, Neuroimaging

## Abstract

Difficulty with reversal learning (RL)—appropriately shifting behavior following outcome contingency changes—may represent a shared or distinct mechanism across eating disorder (ED) diagnoses. We tested whether RL-related neural correlates differ among adults without and with EDs (anorexia nervosa, restricting subtype [AN-R], AN binge-eating/purging subtype [AN-BP], and bulimia nervosa [BN]) and whether these correlates correspond to ED-symptom severity and frequency. We hypothesized that individuals with EDs would demonstrate differential neural activation during RL relative to individuals without, that activation would differentiate AN-BP and BN versus AN-R, and that activation would predict ED-severity metrics. Medically stable participants with AN-R (*n* = 22), AN-BP (*n* = 20), and BN (*n* = 29) comprised the ED group (*N* = 71), contrasted with non-ED controls (*N* = 27). Participants (91% female; M_age_ = 25.9; 80% white, 14.5% Asian) completed clinical interviews and, in a separate session, a probabilistic RL task during functional magnetic resonance imaging. We examined differences in neural activation during RL in the ventral striatum and ventrolateral prefrontal cortex (vlPFC) between the ED and non-ED groups and between diagnostic groups, and conducted exploratory whole-brain analyses. Relations between neural activation and ED symptoms were examined. Lower right vlPFC and ventral striatum activation during RL characterized EDs. No between-ED diagnosis differences emerged. Lower right vlPFC activation predicted more frequent binge eating and purging but not global ED psychopathology. Individuals with EDs may experience difficulty recruiting certain RL-related brain regions, which may relate to difficulty changing ED behaviors. Future directions include investigation of how RL-associated neural networks maintain ED symptoms and influence treatment outcomes.

## Introduction

Anorexia nervosa (AN) is a complex psychiatric disorder that is characterized by dietary restriction, leading to significantly low body weight and disturbance in body weight and shape perceptions (American Psychiatric Association, 2022). Anorexia nervosa is associated with substantial morbidity and up to nine times greater mortality risk than in age-matched controls (Auger et al., 2021). Empirically supported eating disorder (ED) treatments show only small-to-modest efficacy (Monteleone et al., 2022), underscoring an urgent need for novel and specific AN interventions. However, little is known about maintenance mechanisms underlying AN that could serve as targets for such interventions. Additionally, individuals with AN show considerable heterogeneity in symptom expression (Attia, 2010), which may be linked to discrete pathophysiological processes (Der-Avakian & Markou, 2012).

For example, the Diagnostic and Statistical Manual of Mental Disorders, fifth edition (DSM-5-TR) defines two subtypes of AN: restricting (AN-R) and binge-eating/purging (AN-BP) (American Psychiatric Association, 2022). These subtypes are distinguished by the absence or presence, respectively, of two behaviors: (1) binge eating (i.e., eating an objectively large portion of food with a subjective sense of loss of control); and/or (2) purging (i.e., self-induced vomiting or misuse of laxatives, diuretics, or other medications). Maintenance mechanisms differentiating AN-R and AN-BP remain unclear despite some evidence indicating phenotypic and taxonomic differences between the subtypes (Anderluh et al., 2009; Keel et al., 2004). Moreover, there are high rates of diagnostic crossover between AN-R and AN-BP and between AN and bulimia nervosa (BN), an ED characterized by recurrent episodes of binge eating and purging in the absence of low body weight (Eddy et al., 2002; Fichter & Quadflieg, 2007; Tozzi et al., 2005). Thus, identifying both shared and differentiating mechanistic processes underlying AN-R, AN-BP, and BN is important to inform precision approaches to treating overlapping but nosologically distinct ED presentations.

A large body of literature has focused on the neural basis of AN in an effort to identify neuropathophysiological and neurocognitive processes that may drive symptom expression and maintenance. Indeed, meta-analytic data show that AN is associated with aberrant reward, emotion regulatory, and executive functioning circuity that can contribute to altered and overly controlled cognitive and behavioral responses (Su et al., 2021). One of the most well-established neurocognitive correlates of AN is cognitive inflexibility, which refers to an inability to adapt mental processes and behavior to environmental, task, or operation changes (Dajani & Uddin, 2015). Across illness severity levels, adults with AN display greater cognitive inflexibility than non-ED controls, and some deficits persist beyond AN symptom remission or weight restoration (Gura-Solomon et al., 2024; Hemmingsen et al., 2023; Miles et al., 2020). However, cognitive inflexibility is a multidimensional construct, and researchers have long emphasized the need to examine distinct domains of the broader construct to advance research in EDs (Gura-Solomon et al., 2024; Wildes et al., 2014).

Reversal learning (RL)—the ability to alter behavior in response to changes in previously learned reinforcement contingencies (Izquierdo et al., 2017)—is one facet of cognitive inflexibility that has received comparatively less attention in EDs. Reversal learning reflects an evolutionarily adaptive response to environmental stimuli changes whereby one may test behaviors to produce a reinforced outcome or integrate accrued evidence to plan behavioral adjustments that are predicted to be rewarded, and is considered one of the foremost measures of cognitive and behavioral flexibility that has been observed across species (Izquierdo et al., 2017).

In the context of EDs, deficits in RL may reflect an inability to stop previously rewarded ED behaviors (e.g., continuing to engage in purging despite consequences) and thus could help to explain marginal treatment responses and high relapse rates (Dougherty et al., 2024). Additionally, variations in the salience of RL cues and underlying neural activity could help to explain phenotypic heterogeneity within and across AN and BN diagnoses, which has been shown in other psychiatric disorders. For example, variable deficits in RL differentiate bipolar and major depressive or broader mood dysregulation disorders (e.g., where impaired RL is observed in bipolar but not major depressive disorders relative to control participants; Adleman et al., 2011; Dickstein et al., 2010). However, few studies have investigated RL in individuals with AN. There is some evidence of impaired RL in animal models of AN (i.e., rats; Allen et al., 2017). In humans, studies show impaired RL in individuals with AN relative to non-ED controls when assessed by using probabilistic RL tasks (i.e., where selecting the “correct” of two stimuli is rewarded probabilistically rather than continually), with some evidence of greater punishment sensitivity in individuals with AN during these reinforcement paradigms (Geisler et al., 2017; Bernardoni et al., 2018). That is, individuals with AN may be more responsive to negative feedback, which could help to explain disease onset and maintenance.

Despite some overlap with other indices of cognitive flexibility (e.g., attentional set-shifting), the neuro-biological underpinnings of RL are distinct (Izquierdo et al., 2017; Rogers et al., 2000). Meta-analytic data in nonpsychiatric controls point to regions in the reward network, including the ventral striatum (VS) and ventral regions of the prefrontal cortex as central correlates of RL (Yaple & Yu, 2019). More recent evidence similarly implicates ventral striatal and prefrontal cortices in the maintenance of RL processes in human and animal species (Alsiö et al., 2021; Grill et al., 2024; Tavares et al., 2021; Young et al., 2023). The VS and ventrolateral PFC (vlPFC) are respectively linked to prediction errors (i.e., when experienced outcomes differ from expected outcomes) and the accrual of new rules with suppression of previously learned contingencies during RL tasks (Yaple & Yu, 2019).

In adolescents and adults with AN, RL performance has similarly been linked to prefrontal activation in cognitive control, salience, and reward processing regions that include the vlPFC, dorsolateral PFC, inferior frontal gyrus (Hildebrandt et al., 2018), and dorsal anterior cingulate cortex (dACC; Geisler et al., 2017), although one study in recovered women did not find dACC differences relative to non-ED controls (Ritschel et al., 2017). Another study found that learning rates observed during a RL task positively correlated with medial PFC activation while prediction errors positively correlated with VS activation in adolescents and adults with AN (Bernardoni et al., 2018), underscoring that both the PFC and striatum are implicated in aspects of RL in AN.

However, these studies did not differentiate between AN-R and AN-BP; thus, it remains unclear whether neural correlates underlying RL in AN differ by diagnostic subtype, which could inform understanding of unique maintenance processes that can guide precision treatment approaches. We also are unaware of any studies examining neural correlates of RL in BN. Some evidence suggests that elevated ventral striatal and prefrontal gray matter volumes in women with BN (Schäfer et al., 2010) and reduced neural efficiency in frontal cortical learning and reward regions are associated with symptom severity (Donnelly et al., 2018). One recent study found that women with BN who either reported more severe loss-of-control eating relative to other women with BN or felt as though they had binge ate during an in-lab go/no-go eating task demonstrated altered vlPFC activation during the task (Berner et al., 2023), suggesting that symptom expression may be associated with variable neural activation in regions relevant to RL. Finally, recent work by our group utilizing the present sample found that probabilistic RL deficits observed during a behavioral task administered without neuroimaging predicted increased purging frequency for individuals with AN-BP and BN over 6 months but not global ED psychopathology in AN-R, AN-BP, or BN (Dougherty et al., 2024). Together, these findings suggest that deficits in RL may play a distinct role in the progression of AN-BP and BN and relate to the expression of specific ED behaviors (e.g., purging).

Further investigating neural correlates of RL could provide important insights regarding neuropathophysiological processes driving EDs, as well as variations in symptom expression and maintenance across specific ED diagnoses. Thus, the primary aim of this study was to expand upon the results of Dougherty et al. (2024) that utilized the present sample by testing whether neural correlates of RL differ between non-ED controls and individuals with EDs, and whether such correlates differ among individuals with AN-R, AN-BP, and BN. Although understanding of cortical regions of interest in RL continues to evolve (Izquierdo et al., 2017), given evidence in nonpsychiatric controls and limited data in ED samples concordantly implicating the ventral PFC and striatal regions in RL (Yaple & Yu, 2019), we focused on the vlPFC and VS. Specifically, we evaluated the extent to which neural activation in the vlPFC and VS during RL (1) differentiates individuals with EDs from non-ED controls, (2) differentiates ED diagnostic groups from one another, and (3) is associated with ED symptom severity and frequency. We hypothesized that: (1) individuals with EDs would show altered neural activation during the RL task, (2) this activity would distinguish individuals with AN-BP and BN from individuals with AN-R, and (3) the activity would be associated with binge eating, purging, and global ED severity. Finally, we conducted exploratory whole-brain analyses to investigate potential differential activation during RL across ED and non-ED controls and ED diagnostic groups in other cortical regions with a significant whole-brain group effect, as well as associations between activation in these regions and ED symptoms, to identify areas for further study.

## Methods

### Participants

The sample included individuals with AN-R (*n* = 22), AN-BP (*n* = 20), and BN (*n* = 29) and non-ED controls (*n* = 27). Information about this sample has been previously reported (Dougherty et al., 2024). See Table 1 for sample characteristics.

### Measures

#### Structured clinical interview for DSM-5

The Structured Clinical Interview for DSM-5 Axis I Disorders Research Version (SCID-5-RV; First et al., 2015) was used to screen for study exclusion criteria and establish ED and comorbid diagnoses. In the current sample, interrater reliability for psychiatric diagnoses was acceptable (kappa range = 0.61–1.00).

#### Eating disorder examination

The Eating Disorder Examination, 16th Edition (EDE; Fairburn et al., 2008) is a semistructured interview that was used to confirm DSM-5 ED diagnoses and assess ED symptoms. A global score was computed; higher scores indicated more severe symptoms. Additional items assessed frequencies of objective binge eating and purging behaviors during the past 3 months. Internal consistency for the global EDE was high (Cronbach’s α = 0.90). Interrater reliability was high for the global EDE score (ICC = 1.00) and objective binge eating (ICC = 0.93) and purging (ICC = 0.93) behaviors.

#### Probabilistic reversal learning task

**Fig. 1:**
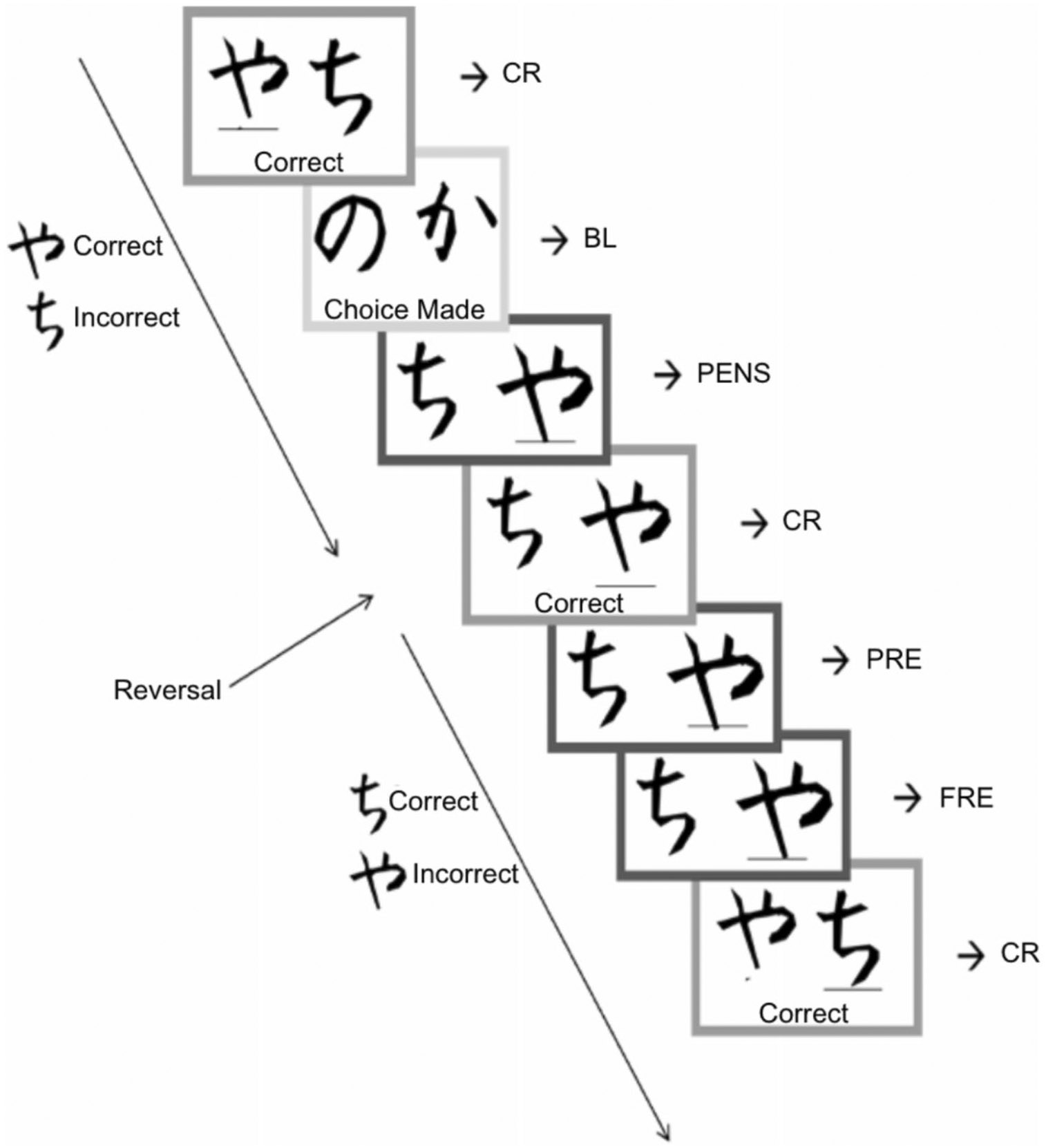
Reversal learning task. Consecutively presented trials where two abstract stimuli are presented on each trial. On baseline trials, participants are instructed which symbol to select and receive neutral feedback (“Choice Made”). On experimental trials, participants attempt to choose the correct of two stimuli and receive positive or negative feedback following their choice. Reversal occurs, without the participant’s knowledge, after six to ten correct responses. CR, correct response; BL, baseline trial; PENS, probabilistic error with no shift; PRE, preceding reversal error; FRE, final reversal error

Neural correlates of RL were assessed using a probabilistic RL task (modeled from Remijnse et al., 2005, 2006) administered during the scanning session. This task consisted of baseline trials, in which no learning occurred, and experimental trials, in which participants learned through trial-and-error using feedback from prior trials. During experimental trials, two abstract stimuli (i.e., Hiragana characters; Frank et al., 2004) were presented simultaneously on a either side of a screen (for 1,200 ms maximally) and participants attempted to choose the “correct” stimulus (i.e., the stimulus more likely to result in positive feedback; Fig. 1). After making their selection, participants were presented with positive or negative feedback (in the form of a 1,500-ms display of the word “correct” or “incorrect” written underneath the abstract stimuli), followed by a fixation cross (jittered between 500 ms, 1,000 ms, or 1,500 ms). Participants received accurate feedback (i.e., the correct response received positive feedback, or the incorrect choice received negative feedback) on 80% of the trials, and they received inaccurate feedback (i.e., the correct response received negative feedback or the incorrect choice received positive feedback) on 20% of the trials. After six to ten correct responses, a reversal occurred (unbeknownst to the participant), such that the previously correct stimulus became the incorrect stimulus. Baseline trials had the same structure as experimental trials, but different stimulus pairs were used, the correct choice was instructed in advance, and feedback was always the word “choice made” (Remijnse et al., 2005, 2006). Baseline trials were not used in the primary analyses. The task consisted of 378 trials, with approximately 90% experimental trials, 10% randomly interspersed baseline trials, and 27 reversals. Trials were separated into three blocks, and different stimulus sets were used in each block.

### Procedure

Study procedures were approved by the University of Pittsburgh Institutional Review Board. All participants provided informed consent prior to participating in the study. Participants were recruited via advertisements, a participant data-base, and referrals from an ED treatment program. Interested individuals were screened via telephone to determine their eligibility for the study. For the entire sample, study inclusion criteria were: (a) proficiency in English and (b) age 18 to 55 years. Exclusion criteria were (a) current psychosis, (b) current bipolar disorder, (c) a severe substance use disorder, (d) daily smoking, (e) a history of neurological disease or concussion, (f) metallic implants, orthodonture, or dental plates, (g) claustrophobia, (h) a full scale IQ score < 80, (i) current pregnancy, or (j) current use of medication that affects cognitive flexibility (e.g., second generation antipsy-chotics). For individuals with an ED, study inclusion criteria were (a) a current DSM-5 diagnosis of AN-R, AN-BP, or BN, and (b) body mass index ≥ 14 kg/m^2^. Exclusion criteria were (a) history of binge eating or purging prior to the onset of AN-R, (b) a history of AN prior to the onset of BN, and (c) current medical instability.

Participants attended two in-person study sessions. During the first session, participants had their height and weight measured and completed clinical interviews assessing psychiatric diagnoses and ED symptoms. During the second session, they completed a 1-h functional magnetic resonance imaging (fMRI) protocol, including neural response to RL. Upon presentation to the second study session, participants practiced the RL task on a desktop computer. Next, they went through familiarization with the scanner environment using a simulator. Lastly, they underwent functional echoplanar imaging scans of blood oxygen level-dependent (BOLD) response during the RL task and a high-resolution, three-dimensional structural scan (6 min). Study sessions were conducted by trained bachelor’s or master’s level research staff who were supervised by licensed clinical psychologists.

#### fMRI data acquisition and preprocessing

Functional neuroimaging data were collected using a 3 T Siemens TIM Trio scanner. Blood-oxygenation-level–dependent images were acquired with a multi-band gradient echo echoplanar imaging sequence (18 slices, three-factor multiband; 2.3-mm isotropic voxels; TR = 1,500 ms, TE = 30 ms; field of view = 220 × 220 mm; matrix 96 × 96; flip angle 58°, bandwidth 1,736 Hz Px–1). Structural 3D axial MPRAGE images (TR = 1,500 ms, TE = 3.19 ms; flip angle 8°; FOV = 256 × 256 mm; 1-mm isotropic voxels; 176 continuous slices) and fieldmaps (2.3-mm isotropic voxels; TR = 550 ms, TE1 = 4.92 ms, TE2 = 7.38 ms; FOV = 220 × 220 mm; flip angle 50°, bandwidth 380 Hz Px–1) were acquired in the same session.

Preprocessing and fMRI image analyses were performed by using Statistical Parametric Mapping software, version 12 (http://www.fil.ion.ucl.ac.uk/spm). For each participant, BOLD images were realigned to the mean volume in the time series and co-registered with the participant’s structural image. Field maps were used to correct for image distortion. Structural images were normalized using a nonlinear transformation to the standard MNI/ICBM 152 tissue probability maps and segmented into gray matter, white matter, cerebrospinal fluid, and other tissues. Blood oxygen level-dependent images were transformed into the same space using the structural image and resampled at 2-mm^3^ isotropic voxel size. Blood oxygen level-dependent images were normalized and spatially smoothed (FWHM 6 mm).

For first-level neuroimaging analyses, a fixed-effect general linear model (GLM) was performed for each participant, with parameter estimates starting with the onset of all trials and modeling the full trial, including response. Trials were classified based on participant response in combination with task stimulus reversals and feedback types (baseline trial, correct response, probabilistic error with no shift, probabilistic error with shift, preceding reversal error, final reversal error, and spontaneous errors). Volumes with high motion were identified by using the Artifact Detection Toolkit (image intensity deviated > 3 standard deviations from the mean intensity or where movement 0.5 mm in translation or 0.01 degree in rotation from the previous image) and were addressed with a GLM regressor. The six realignment parameters were included as regressors. A 128-s high-pass filter and autoregressive modelling were implemented during fitting. Participants who had excessive motion were excluded from analyses. Excessive motion was determined based on having > 20% of volumes with motion > 2 mm and/or 3 standard deviation shifts in signal intensity as determined by ArtRepair (Mazaika et al., 2005). Participants flagged as having excessive motion were removed from analyses (*n* = 2).

### Statistical analyses

#### fMRI data analyses

The following contrast was computed to assess RL: *Final Reversal Errors* > [Probabilistic Errors no Shift + Preceding Reversal Errors] (Remijnse et al., 2005, 2006). Final Reversal Error was defined as the last incorrect response prior to a shift towards the new correct stimulus following a reversal, reflecting learning the new contingencies. Probabilistic Error no Shift was defined as correct responses that were probabilistically given inaccurate negative feedback but did not result in a subsequent shift to the inaccurate response, reflecting behavior based on accuracy rather than feedback. Preceding Reversal Error was defined as incorrect responses immediately after reversal (according to the new criterion) that did not lead to shift to the new correct stimulus, reflecting perseveration or not having updated behavior based on the new contingencies. Thus, this contrast attempts to capture when negative feedback following a reversal is successfully used to shift behavior. To confirm that the RL contrast was associated with increased BOLD activity in the vlPFC and VS, we conducted single sample *t*-tests at each site (Remijnse et al., 2005, 2006). In addition, although not directly relevant to the hypotheses of the present study, we validated the task in accord with previous research (Remijnse et al., 2005, 2006) by conducting single sample *t*-tests for a reward contrast (all positive events > baseline events) in the right VS and for a punishment contrast (all negative events > baseline events) in the insula. The analyses confirmed that the task worked as expected (all *p*-values <.001; Table 2). See the Supplemental Material for detail regarding exploratory whole-brain analyses. Multiple comparisons were corrected using AFNI’s 3dClustSim with the -acf option, applying a voxelwise threshold of *p* < 0.001 and a cluster-level corrected threshold of *p* < 0.05. Regions demonstrating a significant whole-brain group effect included the insula, primary visual cortex, and centromedial amygdala (Table S1; Fig. S1).

#### Primary statistical analyses

Statistical analyses were performed by using SPSS version 29 (ANOVA and *t*-tests) and R version 4.2.1 (regression analyses). Independent samples *t*-tests were used to investigate whether neural activation (i.e., right VS, left VS, right vlPFC, and left vlPFC) during RL differed between individuals with an ED and non-ED controls. A one-way ANOVA was conducted to determine whether neural activation during RL in each brain region differed between ED diagnostic groups (i.e., AN-R, AN-BP, and BN).

Linear regression analyses were used to investigate whether neural activation (right VS, left VS, right vlPFC, and left vlPFC) during RL was associated with ED symptoms (i.e., global ED severity and frequencies of objective binge eating and purging), among individuals with an ED. The model that examined global ED severity included all participants with an ED. The models that examined objective binge eating and purging were restricted to those with AN-BP and BN. All regression models controlled for ED diagnosis, given nuances in symptom presentation and frequency among the ED diagnoses in this study that could drive effects (i.e., with individuals with AN-R not being characterized by binge eating or purging, with individuals with AN-BP being characterized by binge eating and/or purging, and with individuals with BN being characterized by both binge eating and compensatory behaviors such as purging at a minimum set frequency). Given that 12 regressions were run (four brain regions by three outcome variables), the Benjamini–Hochberg False Discovery Rate (FDR) procedure was used to correct for multiple comparisons (Benjamini & Hochberg, 1995). To explore whether any observed associations between neural responses and ED symptoms varied by ED diagnosis, we conducted a planned exploratory analysis adding an ED diagnosis × neural response interaction term to any models that revealed a significant association. Finally, we repeated these regression analyses to investigate associations between neural activation in regions with a significant exploratory whole brain group effect and ED symptoms.

#### Behavioral analyses

To examine whether there were differences in behavioral performance during the task and to isolate differences in neural activation from differences in task performance, we compared choice accuracy (i.e., proportion of correct responses, excluding the first trial after a reversal) between groups. Specifically, paralleling the fMRI analyses, we used an independent samples *t*-test to compare performance between individuals with an ED and non-ED controls and used a one-way ANOVA to compare performance between ED diagnostic groups (i.e., AN-R, AN-BP, and BN).

## Results

### Behavioral performance

There was no statistically significant difference in performance between the ED group and the non-ED control group (*p* =.697), nor between individuals with AN-R, AN-BP, and BN (*p* =.839). See Table 3 for means and standard deviations.

### Group differences in reversal learning neural activation

The ED group showed lower neural activation in the right VS (*p* =.047, *d* =.46) and the right vlPFC (*p* =.041, *d* =.81), but not in the left VS (*p* =.448) or left vlPFC (*p* =.267), compared with the non-ED group. Results of the one-way ANOVAs showed no evidence for differences in neural activation between individuals with AN-R, AN-BP, and BN in the VS and vlPFC (all *p* values >.287). See Table 3 for means and standard deviations. See the Supplemental Material for full results of the exploratory whole-brain analyses. The ED group showed lower neural activation in the insula, *t* = 4.83, *p* <.001, and the primary visual cortex, *t* = 2.12, *p* =.026, and higher activation in the centromedial amygdala, *t* = 4.85, *p* <.001, compared with non-ED controls (Table S3). Results of the one-way ANOVAs indicated neural activation differences between the ED diagnostic groups in the primary visual cortex, *f* = 12.00, *p* <.001, and the centromedial amygdala, *f* = 4.22, *p* =.019. The AN-BP group showed higher activation in the primary visual cortex compared to the AN-R, mean difference (MD) = 1.89, *p* <.001, and BN, MD = 2.31, *p* <.001, groups. The BN group showed higher activation in the centromedial amygdala than the AN-R, MD = 0.29, *p* =.029, and AN-BP, MD = 0.25, *p* =.029, groups (Table S3).

### Associations between reversal learning neural activation and eating disorder symptoms

Across the entire ED group, lower activation in the right vlPFC was significantly associated with a higher frequency of objective binge eating (*B* = − 6.64, *p* =.007, p_FDR corrected_ =.040) and a higher frequency of purging (*B* = − 11.8, *p* =.001, p_FDR corrected_ =.015; Table 4). No other associations were significant. To explore whether the significant negative associations between the right vlPFC and binge eating or purging might differ according to ED diagnostic group, we included group as an interaction term in these models. A significant interaction was found for objective binge eating (*p* =.029), such that for adults with BN (simple slope *B* = − 9.28, standard error = 2.53 *p* <.001), but not those with AN-BP (simple slope *B* = 2.99, standard error = 4.83, *p* =.54), lower right vlPFC activation was related to more frequent binge eating. No interaction with group was found for the negative associations between right vlPFC and purging (*p* =.734). See Table S4 for exploratory whole-brain results. There were no significant associations between activation in the insula, primary visual cortex, or centromedial amygdala and ED symptoms.

## Discussion

This study characterized differences in neural activation in the vlPFC and VS during RL across participants with EDs (AN-R, AN-BP, and BN) and without EDs, as well as associations between neural activation patterns during RL with ED symptom severity. Results indicated greater vlPFC and VS activation in the non-ED group relative to the ED group during RL, consistent with our hypothesis regarding differential neural activation. In addition, exploratory whole-brain analyses indicated that the ED group demonstrated lower insula and primary visual cortex activation, and greater centromedial amygdala activation, during RL relative to the non-ED group. No neural differences emerged across ED diagnostic presentations (AN-R, AN-BP, BN) during RL in the vlPFC or VS. Exploratory whole-brain analyses indicated that participants with AN-BP demonstrated greater primary visual cortex activation relative to participants with AN-R or BN, whereas participants with BN demonstrated greater centromedial amygdala activation relative to participants with AN-R or AN-BP. Lower r-vlPFC activation during the RL task corresponded to more frequent objective binge eating (with this effect being driven by people with BN) and purging, consistent with our hypothesis that neural activation during RL would correspond to severity (i.e., frequency) in these behaviors. Contrary to our hypothesis, neural activation during RL was not related to severity of global ED psychopathology.

Findings demonstrating lower vlPFC and VS activation in participants with versus without EDs is consistent with literature suggesting a potential role for impaired RL in AN (Allen et al., 2017; Bernardoni et al., 2018) and research implicating the VS and vlPFC in maintenance of RL processes (Mitchell et al., 2008; Robinson et al., 2010). Relatedly, our exploratory analyses also highlighted lower insula activation during RL in those with EDs relative to without, consistent with other research suggesting that insula responsivity supports feedback-driven behavior change (Xue et al., 2013). Finally, the relatively lower primary visual cortex activation observed to variable degrees among those with EDs in our exploratory analyses may simply reflect general learning difficulties in this population, as learning is positively associated with response in this region (Furmanski et al., 2004). The present study suggests that people with AN or BN may experience difficulties engaging these areas of the brain in circumstances requiring one to quickly adapt behavior associated with previously learned outcomes. The VS is more specifically implicated in response to reward and the vlPFC is more specifically implicated in reward-related decision making. Findings suggest that individuals with both AN and BN may experience relative difficulties inhibiting previously learned goal-directed actions in situations that require behavioral adaptation to new contingencies and learning from newly established contingencies, perhaps because alternative “adaptive” behaviors may not be experienced as rewarding (i.e., potential VS dysfunction) and/or perhaps because it is difficult to make decisions related to reward outcomes (i.e., potential vlPFC dysfunction). Such difficulties may be important in ED treatment, wherein patients are tasked with changing ED behaviors that may have become relatively automatic over time in the service of prioritizing alternative goals (e.g., eating feared foods, reducing restriction, etc.). Those with EDs may potentially compensate by recruiting other brain areas, as highlighted below.

Results from the present study can be further contextualized with past literature concerned with neural activation during RL in AN. Hildebrandt et al. (2018) reported *increased* prefrontal activation in adolescents with AN during their reversal task phase relative to non-ED controls, which suggested relatively greater engagement of higher-order regulatory pathways when learning from outcomes. These prior findings provide credence to the notion that this type of learning may be more effortful for individuals with EDs. Similarly, Ritschel et al. (2017) found that women recovered from AN demonstrated increased activation in fronto-parietal control areas of the brain specifically in response to negative feedback during RL compared with non-ED controls. Geisler et al. (2017) reported increased dACC activation following negative feedback during RL in adolescent and young-adult patients with AN, potentially suggesting hypervigilance to the impending need to adjust behavior that could be conceptualized as a tendency toward harm avoidance. Indeed, our exploratory analyses showed heightened centromedial amygdala activation in those with EDs that is consistent with literature suggesting amygdala hyperactivity in EDs (Marcolini et al., 2024). This may indicate that the prospect of behavior change may trigger threat-response processes generally across ED presentations, perhaps with some diagnostic heterogeneity in the potency of this effect. These studies, in combination with the results from the present study, suggest that AN may be associated with increased behavioral monitoring and increased involvement of brain circuitry involved in cognitive control during goal-directed behavior, including the ability to make behavioral adaptations involved in RL.

The present findings extend the literature on RL in EDs to highlight that the effortful nature of goal-directed behavior observed in AN may be accompanied by difficulties with response inhibition and learning when outcomes tied to a given behavior change; furthermore, the difficulties identified in this study characterize both AN subtypes and also BN, and thus may represent transdiagnostic mechanisms. We identified no diagnostic differences in neural activation during the RL task when considering our primary regions of interest, contrasting some prior literature that also used fMRI (Murao et al., 2017) but consistent with our past work, which identified no cross-sectional differences between AN-R, AN-BP, and BN on RL task performance in a non-imaging context (Dougherty et al., 2024). Overall, these findings are consistent with a subset of the broader cognitive inflexibility literature suggesting core similarities, as opposed to significant differences, between AN and BN in terms of cognitive inflexibility (Tchanturia et al., 2012). However, we did find that reduced r-vlPFC activation corresponded to greater frequency of ED behaviors (binge eating, purging), with the result for binge eating being accounted for by those with BN rather than AN-BP. Although no prior studies to our knowledge have examined the neural correlates of RL in BN, our results are consistent with some studies that demonstrated reduced neural responsivity to reward in BN (Bohon & Stice, 2011; Frank et al., 2011). Results regarding binge eating are also consistent with a recent study that found reduced r-vlPFC activation during an eating-related go/no-go task corresponded to greater past-month loss-of-control eating among women with BN (Berner et al., 2023). Likewise, this result pertaining to purging is consistent with past work from our group that examined behavioral RL task performance (Dougherty et al., 2024). Finally, in contrast to patterns of findings for neural activation relative to ED behaviors, r-vlPFC activation was not associated with severity of overall ED psychopathology, suggesting a unique role for RL-related neural activation in ED behavior severity (i.e., behaviors occurring more frequently) in AN-BP and BN as opposed to severity of ED cognitive features, such as body-image concerns and dietary rules.

Our findings have several potential implications for research and/or treatment. First, future research might test how changes in neural activation (e.g., increased VS activation, decreased amygdala activation, etc.) during RL correspond to treatment outcomes in AN and BN. For example, changes in neural activation may correspond to more favorable treatment outcomes in obsessive–compulsive disorder (Freyer et al., 2011; Han et al., 2011), a disorder that has notable phenotypic similarity to AN and BN given shared behavioral compulsivity. Explicitly targeting aspects of RL in treatment for AN and BN could correspond to improved neural efficiency that facilitates treatment outcomes given the reduced neural activation during RL observed across individuals with EDs in this study as well as the association between reduced activation and behavioral severity. Future research could also consider if similarities versus differences in neural activation during RL account for diagnostic crossover observed between the AN subtypes as well as from AN to BN. Importantly, it is also worth noting that there is some inconsistency in the literature regarding reward- and punishment-related neural activation in BN and AN (Frank, 2015; Haynos et al., 2020); research capable of providing a more nuanced picture of the neural systems implicated in binge/purge behavior across AN and BN (e.g., employment of tasks with general and disorder-specific stimuli) is warranted.

The use of task-based fMRI to test potential differences in neural activation during RL across multiple ED diagnoses with phenotypic similarities, and between individuals with and without EDs, are notable strengths of this study. Several limitations are also notable. First, our RL task was not designed to interrogate disorder-specific RL, which may be important in examining relationships to aspects of ED psychopathology. Given evidence implicating RL in activity-based anorexia in animal models of AN (Allen et al., 2017), future research could consider examining excessive exercise in relation to neural activation during RL in an ED sample. More generally, given the sample size, the present study may have been generally underpowered to detect differences in neural activation between different ED presentations. Similarly, although there did not appear to be differences in terms of representation of males in the study groups, our total sample of males was small, limiting our ability to examine the role of sex in the present findings. Sex-based differences in neural structure and development are well-documented (Kaczkurkin et al., 2019), and future studies should aim to recruit larger samples of males to ensure both adequate representation and adequate power to detect potential differences. Additionally, we did not account for metabolic or menstrual state, which can influence neural response (Dubol et al., 2021), and thus results should be interpreted cautiously. Finally, white women comprised the majority of the sample, potentially limiting the generalizability of our results to individuals with EDs from other demographics.

## Conclusion

The present results suggest similar neural activation during RL across AN-R, AN-BP, and BN in certain regions associated with reward and reward-related decision making, but differences in neural activation during RL in individuals with EDs compared with individuals without. Considering these results in the context of similar behavioral task performance between those with versus without EDs, learning from changes in outcome-response contingencies may be especially effortful in individuals with EDs and involve recruitment of alternative brain regions to compensate for difficulties.

## Supplementary Material

Supplementary Information

**Supplementary Information** The online version contains supplementary material available at https://doi.org/10.3758/s13415-025-01370-5.

## Figures and Tables

**Table 1:** Descriptive statistics by study group

	AN-R (*n* = 22)	AN-BP (*n* = 20)	BN (*n* = 29)	Non-ED (*n* = 27)
	*n* (%)	*n* (%)	*n* (%)	*n* (%)
Sex				
Male	1 (4.5)	2 (10)	2 (6.9)	4 (14.8)
Female	21 (95.5)	18 (90)	27 (93.1)	27 (93.1)
Race/ethnicity				
White	19 (86.4)	18 (90)	22 (75.9)	19 (70.4)
Asian	4 (18.2)	1 (5)	3 (10.3)	6 (22.2)
Black	0 (0)	2 (10)	4 (13.8)	4 (14.8)
Hispanic/Latino	2 (9.1)	2 (10)	4 (13.8)	0 (0)
American Indian/Alaska Native	1 (4.5)	2 (10)	1 (3.4)	0 (0)
Comorbid diagnosis	11 (50)	13 (65)	17 (58.6)	
	*M* (*SD*)	*M* (*SD*)	*M* (*SD*)	*M* (*SD*)
Age (yr)	27.05 (10.66)	25.4 (6.46)	26.28 (7.93)	26.11 (7.4)
BMI	17.43 (1.15)	17.22 (1.33)	26.09 (4.11)	22.97 (2.61)
ED illness duration (yr)	10.68 (10.17)	10 (6.35)	7.96 (4.61)	
EDE global	2.20 (1.3)	2.86 (1.24)	3.22 (1.04)	0.18 (0.15)
Objective binge eating	0 (0)	29.6 (43.8)	45.72 (36.57)	0 (0)
Purging	0.09 (0.29)	42.05 (62.04)	65.04 (53.95)	0 (0)

Age in the sample ranged from 18 to 52 years. ANOVA indicated that groups did not significantly differ on age (F[3,94] =.142, *p* =.935). Chi-square tests indicated that groups did not significantly differ on sex (χ^2^ = 1.792, *p* =.617), race (χ^2^ = 10.798, *p* =.546), or ethnicity (χ^2^ = 3.741, *p* =.291). Objective binge eating and purging values reflect frequency over the previous 3 months. The most frequent comorbid diagnoses in the total sample were social anxiety disorder (*n* = 19, 19.4%) and major depressive disorder (*n* = 18, 18.4%). ED, eating disorder; AN-R, anorexia nervosa – restricting subtype; AN-BP, anorexia nervosa – binge eating/purging subtype; BN, bulimia nervosa; BMI, body mass index

**Table 2:** Confirmatory analyses of reversal learning contrasts

	*M* (*SD*)	*t*	*p*
Reward contrast: right VS	0.57 (0.78)	7.24	<.001
Punishment contrast: insula	0.34 (0.56)	5.99	<.001
RL contrast: left VS	0.50 (0.91)	5.49	<.001
RL contrast: right VS	0.63 (1.07)	5.84	<.001
RL contrast: left vlPFC	0.84 (0.94)	8.83	<.001
RL contrast: right vlPFC	0.86 (0.82)	10.29	<.001

VS, ventral striatum; RL, reversal learning; vlPFC, ventrolateral prefrontal cortex

**Table 3:** Reversal learning behavioral performance and neural contrast descriptive statistics

	AN-R (*n* = 22) *M* (*SD*)	AN-BP (*n* = 20) *M* (*SD*)	BN (*n* = 29) *M* (*SD*)	*F (p)*	Collapsed ED group (*n* = 71) *M* (*SD*)	Non-ED (*n* = 27) *M* (*SD*)	*t (p)*
Choice accuracy	0.83 (0.08)^a^	0.81 (0.07)^a^	0.82 (0.10)^a^	.176 (.839)	0.82 (0.09)^a^	0.83 (0.09)^a^	.391 (.697)
Left VS contrast	0.34 (0.85)^a^	0.75 (0.86)^a^	0.36 (1.07)^a^	1.271 (.287)	0.46 (0.95)^a^	0.62 (0.79)^a^	.762 (.448)
Right VS contrast	0.40 (1.00)^a^	0.68 (0.90)^a^	0.44 (1.16)^a^	.454 (.637)	0.50 (1.03)^a^	0.98 (1.09)^b^	2.011 (.047)
Left vlPFC contrast	0.71 (0.96)^a^	0.74 (0.55)^a^	0.85 (1.03)^a^	.170 (.844)	0.78 (0.89)^a^	1.01 (1.07)^a^	1.116 (.267)
Right vlPFC contrast	0.71 (0.79)^a^	0.80 (0.56)^a^	0.75 (0.87)^a^	.069 (.933)	0.75 (0.76)^a^	1.13 (0.93)^b^	2.070 (.041)

Different superscripts denote significant mean differences between the collapsed eating disorder and noneating disorder control groups (i.e., an ‘a’ superscript next to both the Collapsed ED group statistics and the non-ED group statistics in the same row indicates the groups did not significantly differ on that row’s variable/contrast per *t*-test results, whereas an ‘a’ superscript next to one group and ‘b’ next to the other indicates the Collapsed ED group and Non-ED group did significantly differ). As indicated by the shared superscripts in each row for the AN-R, AN-BP, and BN columns, no differences were observed between the eating disorder diagnostic groups

VS, ventral striatum; vlPFC, ventrolateral prefrontal cortex; AN-R, anorexia nervosa restricting subtype; AN-BP, anorexia nervosa binge eating/purging subtype; BN, bulimia nervosa; ED, eating disorder

**Table 4:** Associations between reversal learning neural activation and eating disorder symptoms

	*B*	*SE*	*p*	*FDR-corrected* *p*
Left VS contrast				
Global ED symptoms	0.24	0.15	.099	.277
Purging frequency	− 3.38	2.92	.253	.379
Objective binge eating frequency	− 1.88	1.93	.334	.406
Right VS contrast				
Global ED symptoms	0.16	0.14	.221	.378
Purging frequency	− 1.57	2.75	.570	.570
Objective binge eating frequency	1.74	1.80	.338	.406
Left vlPFC contrast				
Global ED symptoms	− 0.10	0.16	.513	.560
Purging frequency	− 5.47	3.26	.100	.277
Objective binge eating frequency	− 2.88	2.17	.192	.378
Right vlPFC contrast				
Global ED symptoms	− 0.29	0.18	.116	.277
Purging frequency	− 11.82	3.43	**.001**	.015
Objective binge eating frequency	− 6.64	2.34	**.007**	.040

Bolded values are statistically significant. VS, ventral striatum; ED, eating disorder; vlPFC, ventrolateral prefrontal cortex

## Data Availability

Data or materials for the experiments are available upon reasonable request, and none of the experiments were pre-registered. Data are not publicly available due to privacy or ethical restrictions.

## Code availability

No custom code was used to generate or process the data described in the manuscript.
