# Supplementary material for: Prefrontal and ventral striatal neural correlates of reversal learning in anorexia nervosa and bulimia nervosa
Source: Cogn Affect Behav Neurosci. 2025 Nov 26;26(3):1316–28. doi: 10.3758/s13415-025-01370-5 (PMC12685067; doi:10.3758/s13415-025-01370-5)
Supplement: Supplementary file 1 — Supplementary file1 (DOCX 5110 KB) [file 13415_2025_1370_MOESM1_ESM.docx]

**Supplemental Material**

**Statistical Analysis of Whole-Brain Data**

Whole-brain voxelwise analysis was conducted using AFNI’s 3dMVM, modeling a single between-subject factor, Group, with four levels (AN-R, BN, AN-BP, HC). No within-subject or random effects were included. Multiple comparisons were corrected using AFNI’s 3dClustSim with the -acf option, applying a voxelwise threshold of p < 0.001 and a cluster-level corrected threshold of p < 0.05. Clusters showing a significant main effect of group were identified for the contrast of interest: Final Reversal Errors > [Probabilistic Errors no Shift + Preceding Reversal Errors; Table S1, Figure S1]. Mean signal estimates from these clusters were extracted using 3dmaskave and analyzed in SPSS and R to examine group-related differences. Task activation was assessed using the intercept for the non-ED control group for comparison purposes with the existing literature (Remijnse et al., 2005) and is reported in Table S2 and Figure S2. To better delineate individual brain regions given the large extent of activation, results are visualized at a more stringent voxelwise threshold of p < 0.00001.

| **Table S1**  *Brain Regions Demonstrating a Significant Whole-Brain Group Effect* | | | | | | |
| --- | --- | --- | --- | --- | --- | --- |
|  |  |  | MNI | | |  |
| Region | Hemisphere | # Voxels | x | y | z | Max-F |
| Insula | R | 21 | 34 | 22 | -2 | 8.88 |
| Primary visual cortex | L | 19 | 0 | -94 | -2 | 8.60 |
| Centromedial amygdala | R | 14 | 16 | -4 | -12 | 9.66 |
| *Note. p* < .001, *p* < .05 corrected; cluster threshold 12 voxels | | | | | | |


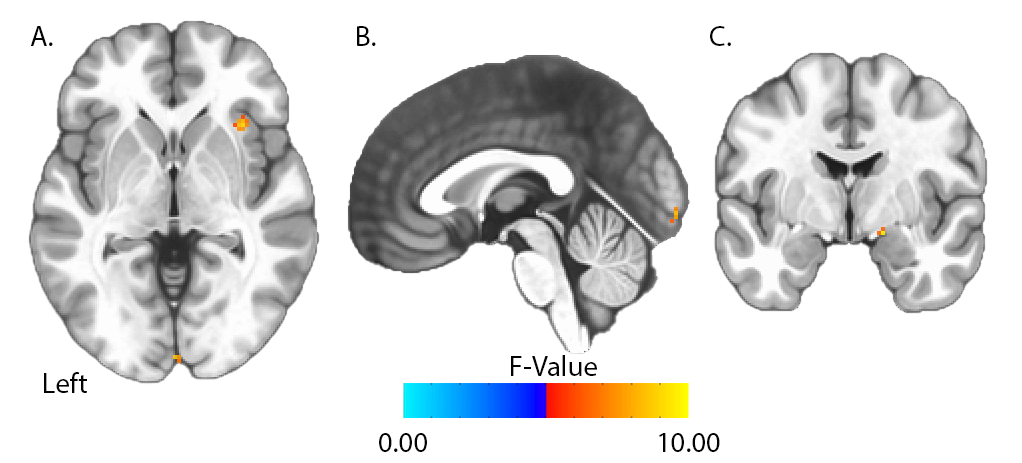
*Figure S1.* Brain regions demonstrating a significant whole-brain group effect: insula (A), primary visual cortex (B), and centromedial amygdala (c).

| **Table S2**  *Task Activation for the Final Reversal Errors vs [Probabilistic Errors no Shift + Preceding Reversal Errors] Contrast Within Non-ED Controls* | | | | | | |
| --- | --- | --- | --- | --- | --- | --- |
|  |  |  | MNI | | |  |
| Region | Hemisphere | Volume | x | y | z | Max-t |
| Supplementary Motor Area | R | 734 | 4 | 20 | 48 | 8.06 |
| Supramarginal Gyrus | R | 657 | 48 | -34 | 42 | 6.86 |
| Inferior Parietal Lobule | L | 654 | -32 | -46 | 40 | 7.61 |
| Precuneus | L | 561 | -8 | -56 | 14 | -7.91 |
| Inferior frontal Junction | R | 440 | 46 | 12 | 36 | 6.31 |
| Anterior Insula | R | 426 | 34 | 22 | -2 | 10.26 |
| Anterior Insula | L | 203 | -32 | 20 | -6 | 6.57 |
| Middle Frontal Gyrus | L | 141 | 32 | 56 | 14 | 6.32 |
| Angular Gyrus | L | 113 | -46 | -72 | 32 | -6.06 |
| Middle Orbital Gyrus | R | 90 | 40 | 56 | -4 | 6.84 |
| Inferior frontal Junction | L | 88 | -48 | 12 | 36 | 5.77 |
| Middle Frontal Gyrus | R | 62 | 44 | 38 | 22 | 5.74 |
| Lingual Gyrus | R | 55 | 8 | -50 | 4 | -5.61 |
| Mid Orbital Gyrus | R | 54 | 4 | 48 | -8 | -5.54 |
| Cerebellum | L | 53 | -10 | -76 | -28 | 5.32 |
| Precuneus | R | 43 | 8 | -60 | 50 | 5.50 |
| Precentral Gyrus | L | 33 | -28 | -2 | 48 | 5.52 |
| Inferior Temporal Gyrus | R | 28 | 44 | -58 | -16 | 5.29 |
| Thalamus | L | 27 | -8 | -12 | 0 | 5.30 |
| Middle Occipital Gyrus | R | 20 | 34 | -66 | 38 | 5.30 |
| *p* < .00001, *p*<.05 | | | | | | |


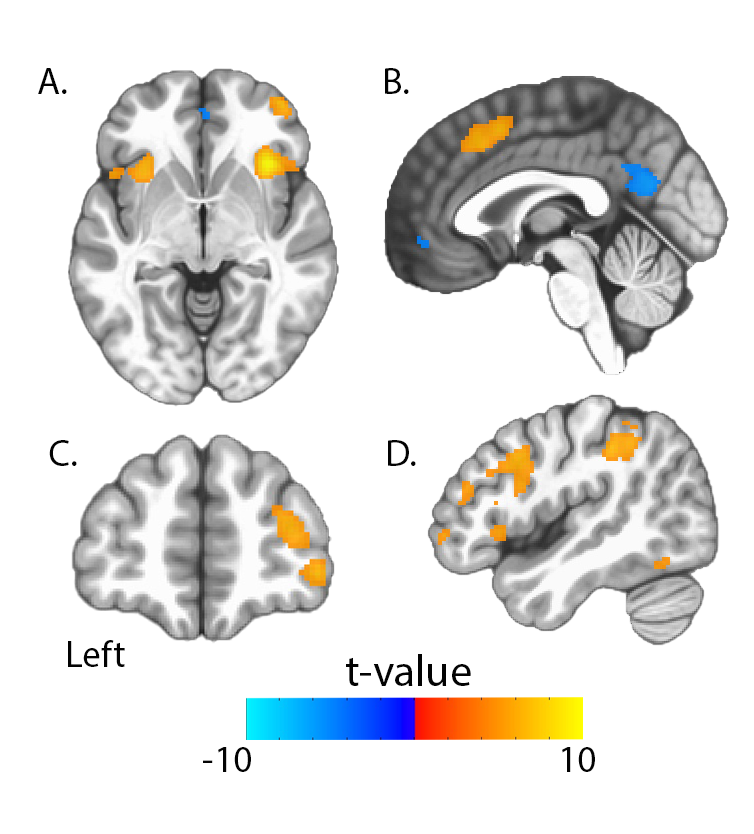


Figure S2. Task activation for the contrast, Final Reversal Errors vs (Probabilistic Errors No Shift + Preceding Reversal Errors). This figure shows activation in the bilateral anterior insula (A), supplementary motor area, mid orbital gyrus, and precuneus (B); middle orbital gyrus, middle frontal gyrus (C), and supramarginal gyrus, inferior frontal junction, middle frontal gyrus (D).

**Table S3**

*Reversal Learning Neural Contrast Descriptive Statistics*

|  | AN-R  (*n* = 22) | AN-BP  (*n* = 20) | BN  (*n* = 29) |  | Collapsed ED group  (*n* = 71) | Non-ED  (*n* = 27) |  |
| --- | --- | --- | --- | --- | --- | --- | --- |
|  | *M* (*SD*) | *M* (*SD*) | *M* (*SD*) | *F (p)* | *M* (*SD*) | *M* (*SD*) | *t (p)* |
| Insula contrast | 0.59 (0.71)^a^ | 0.80 (0.65)^a^ | 0.80 (0.76)^a^ | 0.66 (.520) | 0.73 (0.71)^a^ | 1.65 (1.11)^b^ | 4.83 (<.001) |
| Primary visual cortex contrast | -0.01 (1.13)^a^ | 1.88 (2.06)^b^ | -0.44 (1.73)^a^ | 12.00 <.001) | 0.35 (1.92)^a^ | 1.37 (2.21)^b^ | 2.12 (.026) |
| Centromedial amygdala contrast | 0.97 (0.41)^a^ | 0.11 (0.25)^a^ | 0.36 (0.44)^b^ | 4.22 (.019) | 0.20 (0.40)^a^ | -0.28 (0.52)^b^ | 4.85 (<.001) |

*Note.* Different superscripts denote significant mean differences between the collapsed eating disorder and non-eating disorder control groups, and between the ED diagnostic groups (i.e., an ‘a’ superscript next to both the Collapsed ED group statistics and next to the non-ED group statistics in the same row indicates the groups did not significantly differ on that row’s variable/contrast per t-test results, whereas an ‘a’ superscript  next to one group and ‘b’ next to the other indicates the Collapsed ED group and Non-ED group did significantly differ). AN-R, Anorexia Nervosa restricting subtype; AN-BP, Anorexia Nervosa binge eating/purging subtype; BN, Bulimia Nervosa; ED, eating disorder.

**Table S4**

*Associations Between Reversal Learning Neural Activation and Eating Disorder Symptoms*

|  | *B* | *SE* | *p* | *FDR-corrected p* |
| --- | --- | --- | --- | --- |
| Insula contrast |  |  |  |  |
| Global ED symptoms | -0.28 | 0.20 | .162 | .365 |
| Purging frequency | -9.58 | 3.84 | .016 | .143 |
| Objective binge eating frequency | -5.67 | 2.56 | .032 | .143 |
| Primary visual cortex contrast |  |  |  |  |
| Global ED symptoms | 0.15 | 0.08 | .074 | .222 |
| Purging frequency | 0.23 | 1.57 | .883 | .883 |
| Objective binge eating frequency | 0.27 | 1.03 | .793 | .883 |
| Centromedial amygdala contrast |  |  |  |  |
| Global ED symptoms | 0.11 | 0.37 | .761 | .883 |
| Purging frequency | -6.61 | 7.80 | .401 | .722 |
| Objective binge eating frequency | -3.24 | 5.15 | .532 | .798 |

*Note*. ED, eating disorder.
